# Comparison of Serum and Dietary Selenium Levels in Participants with a Positive History of Recurrent Herpes Lesions and Healthy Individuals

**DOI:** 10.1155/2021/6083716

**Published:** 2021-12-31

**Authors:** Fatemeh Lavaee, Maryam Shahrokhi Sardo, Fateme Zarei, Mahla Shahrokhi Sardo

**Affiliations:** ^1^Oral and Dental Disease Research Center, Oral and Maxillofacial Disease Department, School of Dentistry, Shiraz University of Medical Sciences, Shiraz, Iran; ^2^Student Research Committee, Shiraz University of Medical Sciences, Shiraz, Iran; ^3^Kerman University of Medical Sciences, Kerman, Iran

## Abstract

**Aim:**

In this study, we aimed to compare the level of serum and dietary selenium in participants with a positive history of recurrent herpes labial lesions and healthy controls.

**Materials and Methods:**

This cross-sectional study, conducted during 2020-2021, evaluated the selenium serum level of 40 participants with a positive history of recurrent herpes labial lesions who had referred to Motahhari Laboratory in Shiraz, compared with 38 healthy controls. The selenium level of the serum was assessed by an absorption device, Atomic Graphite Furnace Model FS-240-AAS, made by a US Company. Independent *T*-test was used to compare the selenium level of males and females. In order to assess the mean age value and gender distribution between the two evaluated groups, the independent *T*-test and chi-square test were used, respectively. The serum selenium level was compered between both control and test groups.

**Results:**

The level of serum selenium was not statistically correlated with its dietary level in group 1 (participants with recurrent herpes labialis, *P* value = 0.18) and group 2 (healthy controls, *P* value = 0.6). The serum selenium level was compared between groups 1 and 2, which was significantly higher in healthy controls (*P* value < 0.0001). In contrast, dietary selenium level was not significantly different between patients with a history of herpes labialis and healthy controls (*P* value = 0.48). The level of serum selenium was not statistically correlated with its dietary level in group 1 (*P* value = 0.18) and group 2 (*P* value = 0.6).

**Conclusion:**

Patients with recurrent herpes labialis had lower serum selenium level as compared to the healthy controls.

## 1. Introduction

Recurrent herpes labialis (RHL) is one of the most common oral viral lesions. The most common herpes types in oral lesions are HSV-1 and HSV-2. The former is usually responsible for infections occurring above the waist while the latter is more common in genital viral infections.

Secondary HSV infection can be triggered by stress, trauma, fever, upper respiratory tract infections, and ultraviolet light. Also, immunocompromised people are prone to such infections. One of the necessary elements for human redox system is selenium. Selenium deficiency can increase the risks of some cancers and cardiovascular, neurologic, and infective disorders. According to several in vivo and in vitro studies, selenium supplementation can either decrease or reverse such risks though there are controversial reports in this regard.

Good nutrition, which is the main source for selenium, improves the systemic immunity against infectious diseases. Grains, vegetables, dairy products, seafood, and meat are said to be the richest sources of selenium. People with normal nutritional status can also be susceptible to the mutated virus. Nutritional deficiency, on the other hand, can affect the viral pathogenicity. According to the results of studies on Coxsackie viruses and Influenza, the dietary selenium deficiency under oxidative stress can alter the virus genome and cause more virulent types though the exact molecular mechanism is still unknown. To the best of our knowledge, there is no study on the assessment of selenium deficiency in patients with recurrent herpetic oral and labial lesions, which prompted us to conduct a study aiming to assess the selenium serum level in these patients and compare these values with those of healthy controls.

## 2. Materials and Methods

This cross-sectional study was conducted during 2020-2021 on participants with a positive history of recurrent herpes labial lesions who had referred to Motahhari Laboratory in Shiraz. This study was approved by the ethics committee of Shiraz University of Medical Sciences (IR.SUMS.DENTAL.REC.1399.133). The participants (40 in number) who had reported at least one recurrence of herpetic labials lesions in a year were enrolled in this study. As to the exclusion, pregnant women, participants who had consumed selenium supplements, those with poor nutritional status, and those with an autoimmune disease were excluded from the study. The same exclusion criteria were considered for the control group as well, yielding 38 healthy participants in the control group with no history of recurrent herpetic oral and labial lesions. Finally, those who signed the written concert form were included.

Patients' reception in laboratory was coordinated by assigning a trained dentistry student who attended the laboratory and selected the participants of both groups according to their inclusion and exclusion criteria. She collected the signed written consent forms from the patients, and then, an expert nurse obtained 5 ml blood sample from the participants; 0.5 cc of the sample of patients who fulfilled our inclusion criteria was separated for selenium assessment. The selenium level of the serum was assessed by an absorption device Atomic Graphite Furnace Model FS-240-AAS made by US company Agilent.

The proteins of the samples were deleted by acetonitrile and nitric acid. 0.5 ml of each blood sample was mixed with 0.5 ml of acetonitrile and nitric acid (50 : 50) and shaken vigorously for 5 minutes. It was centrifuged at 3000 rpm. The solution was filtered by special filters with a pore size of 45.0 microns.

**Figure 1:**
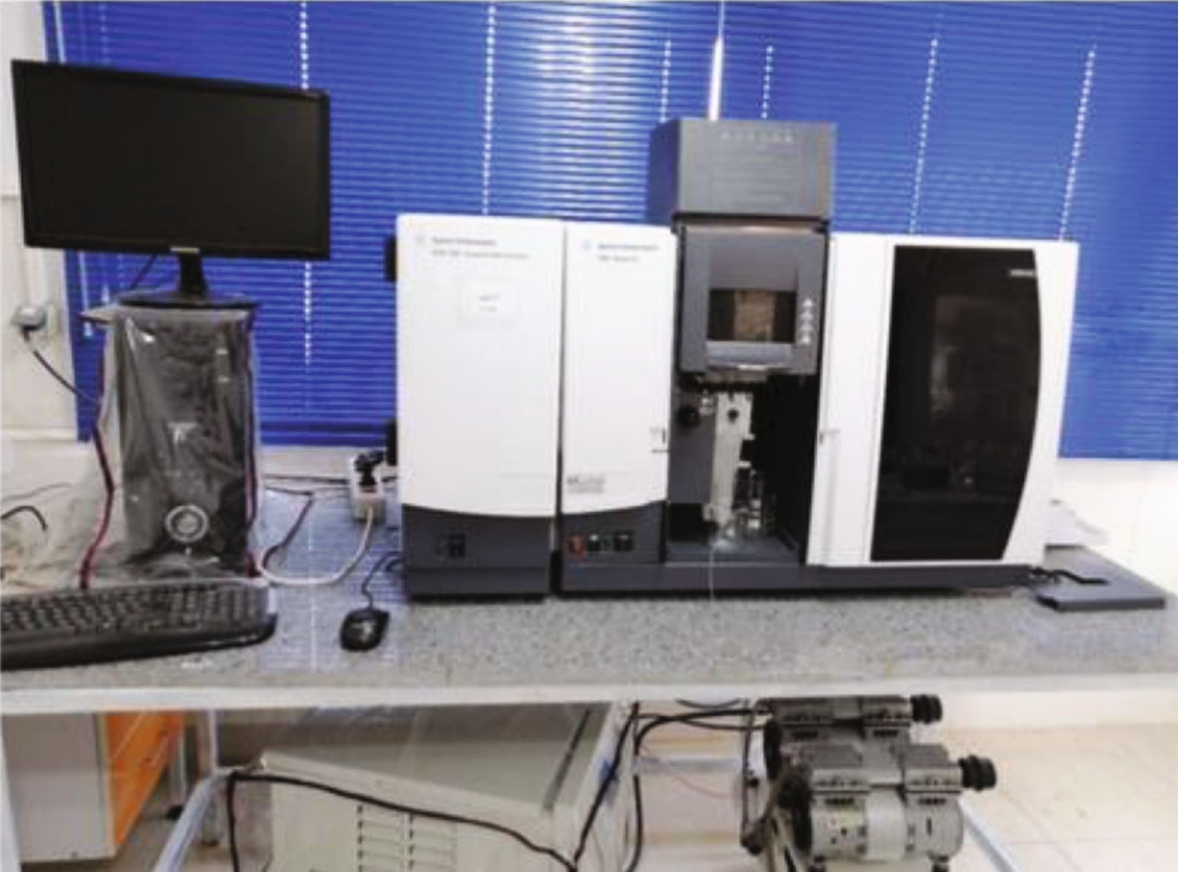
Device Atomic Graphite Furnace Model FS-240-AAS.

We used the absorption device, Atomic Graphite Furnace Model FS-240-AAS made by US company Agilent, for selenium measurement. The atomic absorption device was calibrated by standard selenium samples in the concentration range of 30-200 *μ*g/l. The prepared samples were placed in the autosampler of the device, and the sample concentration was read.  Figure 1 shows the Device Atomic Graphite Furnace Model FS-240-AAS.

To assess the participants' food intake, a nutritionist called them 3 times a week in order to obtain their food intake in 6 meals (6 times per day) during 2 weekdays and one weekend day.

### 2.1. Statistics Analysis

The participants' food content was assessed by Nutritionist IV software; the dietary selenium level was evaluated, and statistical analysis was performed through SPSS version 18. The normality of data distribution has been confirmed by the Kolmogorov-Smirnov test.

Student's *T*-test has been used to compare the age mean value of both evaluated groups. For assessing the mean age value and gender distribution between the two groups, the independent *T*-test and chi-square were used, respectively. The comparison of the serum selenium and diet selenium between control and test groups and also the comparison of the serum selenium level between males and female were done through the independent *T*-test. Finally, Pearson correlation test was used to assess the correlation between the participants' age and serum level.

## 3. Results

Data analysis indicated that the mean age of the participants with a positive history of herpetic lesions (group 1) and that of the healthy controls (group 2) were 40.6 ± 9.36 and 42.67 ± 8.68 years, respectively.

The age distribution in the two groups as seen in Table 1 was not statistically different (*P* value = 0.31).

In group 1, among 40 participants, 34 (85%) were female and 6 (15%) were male. In group 2, among 37 healthy controls, 30 (81.1%) and 7 (18.9%) were female and male, respectively.

According to the chi-square test result, the two evaluated groups were matched regarding gender (*P*value = 0.64) and the age of participants.

The difference between the serum selenium level of groups 1 and 2 was significant; it was higher in the healthy controls (*P* value < 0.0001). In contrast, the selenium dietary level between patients with a history of herpes labialis and healthy controls was not significantly different (*P* value = 0.48). The result also showed that the level of the serum selenium was not statistically correlated with its dietary level in neither groups (group 1: *P* value = 0.18 and group 2: *P* value = 0.6).

Table 2 shows the mean value of selenium serum and dietary level in both groups.

The correlations between serum selenium level and dietary selenium level and age are displayed in Table 3. As the table shows, there is no correlation between age and selenium, in neither serum nor dietary level, in groups 1 and 2.

The mean of the participants' selenium level regarding gender was reported in Table 4. As seen in the table, there was no significant difference between males and females in neither group.

## 4. Discussion

According to the results of the current study, the mean serum level of selenium in patients with a history of recurrent herpes labialis was lower than that of healthy controls. To the best of our knowledge, there is no similar study about recurrent herpes labialis since the effect of selenium deficiency has been assessed on different risk factors of some other viruses such as HIV [1], COVID-19 (coronavirus disease) [2, 3], Coxsackie virus (CV) [4, 5], Influenza virus [6], HCV [7], porcine circovirus Z (129), and cytomegalovirus [8].

In accordance with our study results regarding selenium deficiency in patients with recurrent herpes labialis, Majeed et al., in an assessment conducted in 2021, found a significantly lower serum selenium level in patients with confirmed COVID-19 infection in India [2].

Few studies have revealed the relationships between selenium deficiency and HIV susceptibility and progression. Lower CD4 count in HIV patients with suboptimal serum selenium level, which can induce more HIV progression and higher death rates, has been reported [9–11]. The selenium deficiency has been so important that it has been introduced as a predictor for HIV patients' survival [10].

However, no significant difference has been reported between plasma levels of selenium in HIV positive and negative participants in Watanab et al.'s study [1, 12–15].

For example, a number of studies have confirmed daily supplementation of selenium in HIV-infected patients, which has been effective on disease progression suppression [16, 17].

However, another study found that selenium supplementation had no effect on viral load reduction in a Danish population [18].

Erkekoğlu et al. reported lower plasma selenium in children infected with highly pathogenic H1N1 subtype of Influenza A virus [19].

Selenium supplementation in randomized controlled trials showed better humoral response following Influenza A vaccination in comparison to the control group [20].

There is evidence of the necessity of selenium supplementation prescription for different patients.

In an animal study, it was shown that selenomethionine and selenium supplementation inhibits porcine circovirus Z, which is a DNA virus from the Circoviridae family [21–26]. The antioxidant activity of selenium induces such an effect and may finally reduce inflammation [22]. Other studies on DNA viruses with different methods are briefly reviewed in the literature.

In an evaluation, GPX reduction has been associated with viral load increment [8].

Selenium supplementation induces faster healing of viral lesions including HSV2 (herpes simplex virus 2) [27], human herpes virus 3 (HHV3) [28], cytomegalovirus [8], and oral human papillomavirus (HPV) [27, 29]. In addition to viral load, reduction in different antiviral cytokine increments after selenium supplementation has also been reported.

Selenium deficiency can shift TH1/TH2 balance toward higher level of Th2 phenotype (IL4 cytokine), while this balance can shift toward TH1 phenotype (IL2, INF-*γ* cytokine production) in cases with selenium supranutrition [30, 31].

Selenium status activates macrophages conversely. Dietary selenium supplementation in selenium-deficient people may support the proinflammatory cellular immune reaction (Th1 type) against viral infection; however, the immunity reaction should be prevented from excessive Th1 activation by managing the macrophages toward more anti-inflammatory phenotype [30].

Selenium supplementation increases the T cell proliferation and natural killer cell activity, which can enhance cellular immune reaction in selenium-deficient people, but high dose of selenium supplementation may have some side effects [32] including selenium toxicity [33, 34], alopecia [35], dermatitis [35], increased mortality [36, 37], and type 2 diabetes [37] and increase the risk of prostate [38] and nonmelanoma skin cancers [39]. The imbalance between reactive oxygen or nitrogen species production and the protective effect of antioxidants is considered as oxidative stress. The antioxidant system has different defense levels with the major forces being on prevention and removing free radical generation.

One of these antioxidant enzymes is named glutathione peroxidase (GPX). Glutathione peroxidase 1, 2, 3, 4, and 6 (GPX) contain selenium in their active structural sites and are responsible for hydrogen peroxide reduction catalyzing for cellular protection from oxidative damage [40].

In our study, the dietary mean value of selenium was not statistically different between the two groups, which means the comparison of their serum level is reliable.

To the best of our knowledge, there are different ranges of normal serum selenium level. Such diversity in normal range is presented in different studies. Different diets and dietary cultures in addition to the soil selenium level can affect serum selenium. Also, protein-rich foods such as sea protein (fish, shrimp, etc.) are the richest source of selenium. According to the literature, serum selenium lower than 80 mg/l denoted deficiency [41].

In this study, participants with a history of herpes labialis had a low serum selenium level as compared to the normal range. This can prompt future studies.

Selenium supplementation for susceptible patients may trigger the herpes virus activity and lead to its recurrence. In addition, evaluation of the herpes labialis recurrence and its relationship with serum selenium or recurrence rate is worth further investigating. Since the number of females and males was not matched in our study, we did not compare the mean serum selenium with regard to gender. Therefore, a bigger sample size is suggested for more precise evaluation. In the present study, assessing the dietary selenium made the serum level comparison more precise.

## 5. Conclusion

The level of serum selenium was not statistically correlated with its dietary level in group 1 (*P* value = 0.18) and group 2. There was no significant difference between males and females in both groups regarding the selenium level. There was no correlation between age and selenium in neither serum nor dietary level, in both groups. Finally, we found that patients with recurrent herpes labialis had low serum selenium level in comparison to healthy controls.

## Figures and Tables

**Table 1:** The age distribution in group 1 and group 2.

	Group	*N*	Mean	Std. deviation
Age	1.0	40	40.6	9.36
	2.0	37	42.67	8.68

Group 1: positive history of herpetic lesions; group 2: healthy controls.

**Table 2:** The mean value of selenium serum and dietary level and their comparison between group levels.

Group	1		1		2		2	
	Serum selenium (ppb)		Dietary selenium (mg)		Serum selenium (ppb)		Dietary selenium (mg)	
Sex	Women	Men	Women	Men	Women	Men	Women	Men
*N*	32	6	25	3	30	7	27	7
Mean	76.91	65.57	0.05	0.04	115.83	103.67	0.05	0.06
Standard deviation	18.84	25.22	0.019	0.02	19.09	9.64	0.02	0.02
*P* value	0.20		0.36		0.11		0.38	

Group 1: positive history of herpetic lesions; group 2: healthy controls.

**Table 3:** The correlation of serum selenium level and dietary selenium level and age.

		Age group 1	Age group 2
Serum selenium (ppb)	Pearson correlation	-0.041	-0.212
	*P* value	0.8	0.2
	*N*	38	37
Dietary selenium (mg)	Pearson correlation	0.01	0.13
	*P* value	0.95	0.46
	*N*	28	34

Group 1: positive history of herpetic lesions; group 2: healthy controls.

**Table 4:** The mean level of the selenium of participants according to gender.

		Group 1			Group 2		
	Sex	*N*	Mean	Std. deviation	*N*	Mean	Std. deviation
Selenium (ppb)	Women	32	76.91	18.84	30	115.83	19.09
	Men	6	65.57	25.22	7	103.67	9.64

Group 1: positive history of herpetic lesions; group 2: healthy controls.

## Data Availability

The readers can access the data supporting the conclusions of the study by a request through an email to the corresponding author.
